# Wind Farms and Power Lines Have Negative Effects on Territory Occupancy in Eurasian Eagle Owls (*Bubo bubo*)

**DOI:** 10.3390/ani12091089

**Published:** 2022-04-22

**Authors:** Magne Husby, Martin Pearson

**Affiliations:** 1 Section of Science, Nord University, 7600 Levanger, Norway; 2 Odontovet, Nedre Vikan 5, 7240 Hitra, Norway; martin@odontovet.no

**Keywords:** anthropogenic disturbance, birds, construction, influence area, territory, tolerance, turbines, wind energy, wind farm construction

## Abstract

**Simple Summary:**

Wind power can contribute to a necessary reduction in CO_2_ and other greenhouse gas emissions. However, wind farm construction and infrastructure might cause other problems, for example, reducing biodiversity. In parts of their distribution area, eagle owls are scarce and declining, and not much is known about their tolerance for different kind of disturbances. Here, we investigated the presence–absence of Eurasian eagle owls (*Bubo bubo*) in 48 territories in the central part of Norway before the construction of eight wind farms and power lines started, and shortly after the construction period. Eagle owls living within 4–5 km away from the disturbance left their territories to a higher extent than eagle owls living even further away.

**Abstract:**

Wind power is useful for reducing greenhouse gas emissions, but the construction and operation might have negative effects on biodiversity. The purpose of this study was to investigate any effects of wind farm and power line construction on territory occupancy in the vulnerable Eurasian eagle owl. We investigated 48 eagle owl territories before and after the whole construction period and a short operation period with the use of sound meters. We found that territorial eagle owls within 4–5 km from the wind farm and power line construction disturbance left their territories to a significantly higher extent (41% reduction in the number of territories with eagle owls) compared with the eagle owls in territories further away (23% reduction). The distance from the nest site to the disturbance was significantly shorter for those territories that were abandoned compared with territories where the birds stayed. Possible reasons for this decline might be a higher mortality caused by collisions, desertion and avoidance of wind power areas caused by the noise and disturbance from their construction. In addition, there are possible indirect effects, for example reductions in prey species may force eagle owls to abandon their territories. The construction period lasted much longer than the period with active wind turbines and power lines in this investigation, but we cannot separate the effects of the two because the investigations were only possible in the eagle owl breeding season, and the wind turbines were activated shortly after the construction period. Our results imply that careful investigations are needed to detect the possible occurrence of eagle owls near any type of construction work. Studies of these territories should strongly influence how and when the construction work can be carried out, but more investigations are needed to find details about the influence of distance.

## 1. Introduction

It is unequivocal that human influence has warmed the Earth’s atmosphere and oceans more in the last 50 years compared to the last 2000 years, causing many weather and climate extremes worldwide. Strong reductions in CO_2_ and other greenhouse gas emissions in the coming decades are needed [1]. The adverse effects of climate change on biodiversity are expected. With a global temperature increase of 1.5–2 °C, the majority of terrestrial species ranges are projected to dramatically shrink [2]. Many bird species have already experienced declines caused by global warming [3,4].

Wind power is one of several possible mitigation actions to reduce greenhouse gas emissions [5]. However, much of nature has already been lost, and what remains continues to decline. Now, only 23% of global land area is classified as wilderness [2]. Land-based wind farms require huge areas, and this effort to reduce global warming might increase biodiversity losses. Investigations have shown the negative effects of landscape disturbance and land use on many bird populations [6,7,8], including boreal owl (*Aegolius funereus*) and northern saw-whet owl (*Aegolius acadicus*) in Canada [9]. More specifically, the construction and operation of wind farms negatively impacts birds both by habitat alteration and disturbance [10], as well as direct mortality [11,12,13,14]. The fatality rate due to wind turbines is relatively high for some owl species compared with some other bird species [15]. Eagle owl mortality has been associated with both wind turbines [16] and power lines [17,18,19,20,21,22].

During wind farm construction, strong anthropogenic noise is likely an important disturbing factor for birds in the surrounding areas [23,24,25,26,27]. Farmland birds decline more significantly near urban areas compared with rural areas with less anthropogenic impact, including noise [28]. Owls are, to a large extent, acoustically specialized predators, and therefore potentially vulnerable to noise. The morphology of eagle owl wings makes it possible to fly almost silently [29], as an adaptation to finding prey by listening while flying. Anthropogenic noise was found to reduce the hunting success of northern saw-whet owl (*Aegolius acadius*) by 8% for each decibel increase in the noise [30]. Helicopter overflights caused Mexican spotted owls (*Strix occidentalis lucida*) to flush when the distance was less than about 105 m, but there were no effects on reproductive success or the number of fledglings. Chainsaws were found to be more disturbing to this owl species than helicopter flights at comparable distances, but there was still no visible negative effect beyond 105 m [31]. Noise from low-intensity chainsaws operated at an 100 m distance from roost sites did not elicit a detectable increase in physiological stress levels in California spotted owls (*Strix occidentalis occidentalis*), but chronic and intense noise from for example road construction was not included in the experiment [32].

Human activity increases in remote areas during the period of wind farm construction. It has been found that Mexican spotted owls leave their roosting site when approached by hikers, but mostly when the hikers were within 55 m [33]. Breeding females of the Mexican spotted owl decreased the amount of time spent handling prey and daytime maintenance during experimental hiking. Therefore, the authors concluded that the cumulative effects of high levels of short-duration recreational hiking near nests may be detrimental [34].

Very little is known about the effect of human presence on Eurasian eagle owls. Roosting eagle owls are not especially shy [35,36,37], and flush distances of 50 m or less are observed [35]. However, in parts of the area used in the present study, an increase in the number of hikers within 2 km from the nest site reduced eagle owl breeding performance [38]. Eagle owls might be more sensitive to approaching humans in areas where they have been heavily persecuted than in other areas. High rates of persecution during more than hundred years is one important factor resulting in declining eagle owl populations in Norway [18,36,37]. The eagle owl population in Norway is estimated at 451–681 pairs [39], classified as endangered (EN) on the Norwegian red list [40].

Other investigations of eagle owl found that the number of breeders declined when the number of hikers and climbers increased in a national park in Croatia, but the number of pairs investigated was low [41]. In 327 clutches studied for 20 years in Bulgaria, human activities near the nests were the main reason for nest failures [42]. In the Italian Alps, territories were located at a lower elevation and closer to intensively cultivated, urbanized valley floors where there was more prey available, but the eagle owls on the valley floors suffered a higher anthropogenic-associated mortality [43].

The present study investigates the effects of the noise and other disturbance on eagle owls in Norway during the construction of both wind farms and power lines, and a short period with active wind turbines. We investigated areas with eagle owls before the disturbance started and in the first breeding season after the wind turbines were activated. According to the literature introduced above, we expected to (1) find fewer occupied eagle owl territories after the construction period than before the construction started within the influence areas compared with reference areas further away from the disturbances. In addition, (2) we expected a lower breeding performance within the influence area compared with the reference areas.

## 2. Materials and Methods

### 2.1. Study Area 

**Figure 1:**
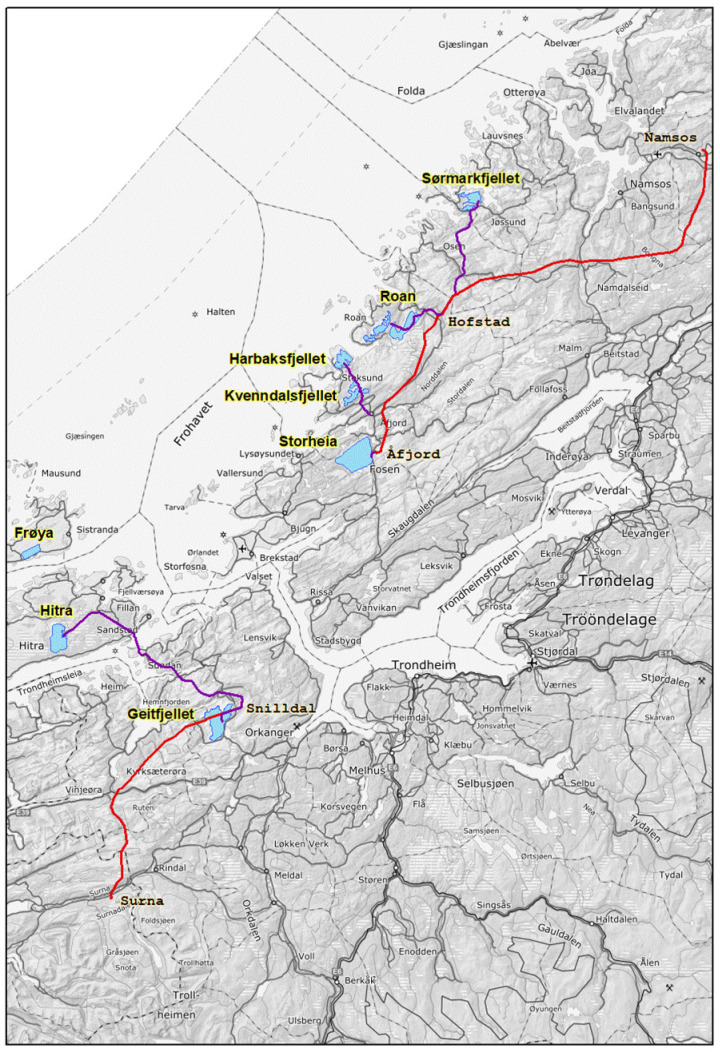
The study area with the eight wind farms (blue) with area names (yellow), and the 420 kV (red) and 132 kV (violet) power lines. The influence area for eagle owls is defined to be up to five kilometers from these constructions, and reference areas are further away. Nearly no new powerlines were constructed in Frøya.

The study area comprises eight wind farms and associated power lines in the central part of Norway (Figure 1). In 2014–2015, we investigated 70 areas known for having eagle owls commonly recorded or where eagle owls might have been recorded during the last decade. This is the pre-disturbance investigation. The home ranges of the eagle owls vary considerably between areas, likely as a response to variable food supply, sex and season [18,35]. Some individuals might have home ranges of several tens of km^2^ [18,35,44,45,46]. They also hunt outside of the strictly defended area [35]. Therefore, we started this investigation by defining all areas within 5 km from the wind farms or power lines as belonging to the influence area (Figure 1), and other areas to be outside the influence area and used as reference areas. However, in this study, we investigated at which distance we still observed the negative effects of the constructions. All areas included in the investigation were close to the coast, and the influence areas and reference areas were situated in the same region (the wind farm areas and the areas around and between them in Figure 1) and are therefore comparable. 

Parts of the study area are mountains without forests and suitable for wind farms, with steep cliffs that are the preferred breeding ground of the eagle owl. In addition, there are forests, farmland areas, bogs, lakes and rivers, and some human settlements. Islands with breeding eagle owls in the study area lack the mammalian predators common on the mainland, such as the red fox (*Vulpes vulpes*), Eurasian lynx (*Lynx lynx*), European badger (*Meles meles*) and pine marten (*Martes martes*). The eagle owl diet analyzed from pellets, remnants found on and near the nesting cliffs of our study area show a wide variety of prey, especially various species of birds, mammals and reptilians [47,48,49,50]. This is also normal also in other areas of its breeding range [18].

### 2.2. Construction Disturbance

The disturbance to eagle owls investigated in the present study are mainly in the construction period of the wind farms and power lines, both power lines connected to the wind farms and other new power lines constructed in the same time period. In addition, the areas have more human activity than before.

The main disturbances in connection with wind farm construction are supposed to be road construction and the construction of platforms for wind turbines, transport and installation of the turbines with the use of cranes and large trucks. Disturbance along the power line network involves clearing a belt free of trees in the power line ride by using logging machines or chain saws; helicopters are used to transport materials and to install the electric lines. Normally several rig areas and storage spaces are constructed along the power lines. Mechanical diggers and dynamite are used in the infrastructure construction. There were no restrictions in where and when construction work was permitted, except when eagle owl were detected within about 1 km from the working area. As a result of our findings in 2014–2015, before the construction period started, a few wind turbines and roads were moved relative to the original plans to reduce the disturbance of neighboring eagle owl nests or possible nests.

During the wind farm construction period, there was more human activity than usual in the remote areas, but this was not quantified. That, together with the construction disturbance, most likely reduced the preferred habitats for the eagle owls and for some of the prey species. After the construction period, the wind turbines started to produce electricity and generated a different but significant type of noise. The disturbance in the influence areas lasted, therefore, 2–3 years during the construction period and a few months with active wind turbines and power lines. It was impossible for us to start the investigations immediately after the construction period because we had to wait until the first breeding season afterwards. After construction, the power lines might also have caused mortality by collisions [22].

### 2.3. Observing Eagle Owls

We used wildlife acoustic sound meters (SM 2+, SM4 and SM Mini), in 2014–2015, programmed to continuously record for about seven days in March (February–April), termed the spring investigation. In 2020–2022, the sound meters were programmed to record from one hour before sunset to one hour after sunrise for about 14 days, thus increasing the probability of detecting eagle owls if present. In areas where the eagle owl was not registered in the spring investigation, we used recorders in the autumn (September), similarly programmed according to sunset and sunrise as in the spring. Three localities were investigated in February–March 2022. The autumn investigation is of course not completed yet, but our experience is that there will be almost no eagle owls registered in the autumn if they were not present in the breeding season. Different areas were investigated in different years, and each area was investigated only one year before the disturbance and one year after the disturbance period.

The recordings were analyzed by the programs Audacity(R) editing software (v. 2.4.2. Boston, MA, USA) and Kaleidoscope (Pro Analysis Software v. 5.1.9g, Wildlife Acoustics. Maynard, MA, USA) to find eagle owl sounds, and Raven (Pro v. 1.6, Cornell Lab of Ornithology. Ithaca, NY, USA) for studying the details. The song of the eagle owl is not learned; therefore, it has little variation over time [51]. It is therefore possible to recognize different males via variations in spectrogram measures if the recordings are high-quality [52,53,54]. We used the program Raven to find details in the song of males to quantify the differences, and thereby concluded if it was the same or a different male in neighboring areas, treating the areas as one territory if it was the same male.

In addition, we measured the distance from the nest, or the most probable nesting place, to both the closest wind turbine and the shortest distance to a new power line constructed after the preliminary investigation. We are not sure whether or not every area with observations of eagle owls was a breeding territory, but because most areas had regular observations of eagle owls during the years before this investigation started, we considered all the areas as territories.

### 2.4. Statistics

To test hypothesis 1, if the eagle owls changed their presence status in the territory from 2014–2015 to 2020–2022, we used generalized linear mixed effects model (GLMM) analysis (IBM Statistics v. 27. Chicago, IL, USA). Alternatives were 1 = abandoned (*n* = 14); 2 = no change in observation, meaning observed in both periods (*n* = 23); and 3 = observed only in the last period, termed reestablished (*n* = 2). Instead of reestablishment, it is possible that the eagle owls used the territories in 2014–2015 without being detected, but statistically, we treated these territories as reestablished. Explanatory variables were: (1) inside or outside the influence area, varying from 1 to 5 km (values 1 and 2, respectively); (2) distance between the nest area and the closest wind turbine; and the (3) shortest distance between the nest area and a new power line. Both distances were measured to the nearest 0.1 km. Each wind turbine and the powerlines are visible on norgeskart.no, which has a tool for measuring distances. In addition, (4) we included the island and mainland as explanatory variables with values 1 and 2, respectively. The observations in the different territories were not in the same year, and year was therefore included as random factor. The GLMM analysis was run with a multinomial probability distribution and cumulative logit link function.

In the data exploration for the GLMM analysis with the target variable, if eagle owl changed their presence in the territory or not from 2014–2015 to 2020–2022, we first used Spearman rank correlations between the explanatory variables. We used the variables for: (1) within or outside the influence area, (2) the distance from the nest area to the nearest wind turbine, and (3) the closest distance to the power line. The correlations were quite high (r_s_ = 0.6–0.8) and around the suggested maximum limit of 0.7 [55]. The variation inflation factor (VIF) values were >5.5, which were above most recommendations [56,57]. We therefore run the GLMM analyses with the island, mainland and only one of the other explanatory variables that were highly correlated separately (distance from nearest wind turbine, distance to power line, within or outside the influence area) and by varying the influence area from 1 to 5 km from the nearest turbine or power line. We compared the different models with Akaike information criteria corrected (AIC_C_), and with ΔAIC_C_ > 2 from the best model, the other models were normally rejected [56]. 

Because of the small number of reestablished territories, we ran a nonparametric Fisher–Freeman–Halton exact Test (FFHET) to test if the decline in the number of active territories was statistically significant with various influences of distance.

We tested hypothesis 2 with a GLMM analysis using only nests with known breeding performances for all the seven years from 2015 to 2021, produced fledglings in at least one of the seven years. The year 2015 was before the actual disturbance started and 2021 was the year after. The target variable was a breeding performance ranked from 1–6: 1 = eagle owls were not observed in the territory, 2 = observed in the territory, 3 = eggs were laid, 4 = one chick was produced, 5–6 = one additional point for each additional chick. Chicks produced is the number of young alive at ringing age during the first 15 days of June, about three weeks old. Explanatory variables were: (1) islands Hitra and Frøya, values 1 and 2, respectively; (2) disturbance with value 1 in the years without disturbance, and value 2 in the years with disturbance; (3) distance to the nearest disturbance factor (wind turbine or power line) in km; and (4) year (during the seven year period, 2015–2021). The GLMM analysis was conducted with multinomial probability distribution and cumulative logit link function. The sample size was relatively low in this analysis, but with the selected limitations, we were sure that the adult birds were living in a territory where reproduction was possible. No explanatory variables were excluded because the maximum values of the correlations between the explanatory variables were within the recommended limits (|r_s_| < 0.46 and VIF < 2.2). Territory number was a random factor in these analyses.

GLMMs were used because they removed variability in responses that were associated with random factors rather than the conditions of experimental interest, thus reducing Type I error rate [58]. GLMM may be the best tool for analyzing non-normal data that involve random effects [59]. Because of the strong probabilities of negative effects of the disturbances, statistical tests are one-tailed with an *α*-level of 0.05.

## 3. Results

Of the 70 areas investigated in 2014–2015, 22 were excluded from further investigations due to the lack of eagle owl activity in 2014–2015 and earlier registrations were scarce and/or several years old. In the remaining 48 territories, we registered eagle owls in 37 territories, and continued to investigate the other 11 territories despite the fact that no eagle owls were observed in 2014–2015. This is because the potential for reestablishment/detection was expected to be higher here than in the 22 territories that we excluded according to the earlier history of the locations. In 2 of these 11 areas, eagle owls were registered in 2020–2022. Therefore, we had 39 eagle owl territories with registrations in at least one of the two time periods that were included in the analyses.

Before the construction period started, we observed at least one eagle owl in 15 territories within the influence area of 5 km from the closest wind turbine or power line and 22 territories outside the influence area. Of the territories within the influence area, nine were abandoned. In addition, two territories within the influence area with no eagle owls detected before the construction period were detected afterwards. We therefore registered seven fewer territories of the 17 with eagle owls in at least one of the two periods (41% reduction). Outside the influence area, five of the 22 territories were abandoned, and there were no reestablished territories (23% reduction). The decline in the number of active territories was statistically significant both with an influence distance of 5 km (FFHET value = 7.39, *p* = 0.008) and with an influence distance of 4 km (FFHET value = 4.97, *p* = 0.028). The other tested influence areas from 1 to 3 km did not yield significant results in the same test (*p* > 0.15 in all tests).

**Figure 2:**
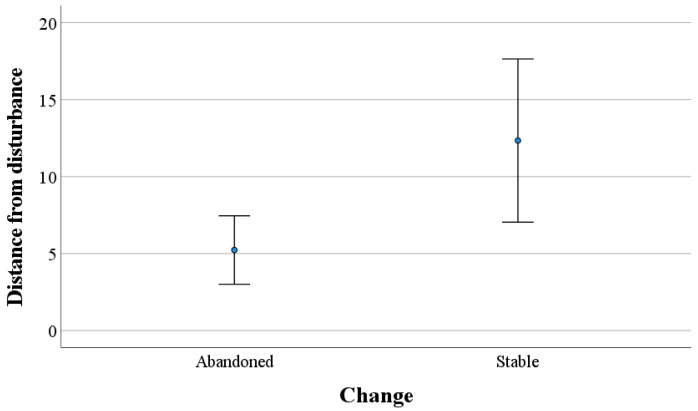
Mean distance (km) ± 2 SE between the central places of eagle owl territories or nest sites that were abandoned (*n* = 14) or no change in occupation (*n* = 23) and distance to closest disturbance (wind turbine or power line). The change is a comparison of territory occupancy before the construction of the wind farms and power lines started compared with the similar investigation shortly after the construction was finished.

Abandoned eagle owl nests (*n* = 14) were significantly closer to the nearest disturbance source than those that remained (*n* = 23) (MW U-test: Z = −1.817, *n* = 37, *p* = 0.035) (Figure 2). We found a similar result for the distance from power lines (MW U-test: Z = −1.645, *n* = 37, *p* = 0.050), and the results were not so significant for the distance to the nearest wind turbine (Z = −1.472, *n* = 37, *p* = 0.071). The two areas where eagle owl reestablished were 2.5 and 4.0 km away from the nearest wind turbine, respectively.

None of the GLMM analyses testing hypothesis 1 were statistically significant. An influence distance of 4 km gave the best model, as judged from AIC_C_ values, and all the other variables achieved a value of ΔAIC_C_ > 2 compared with the best model.

**Figure 3:**
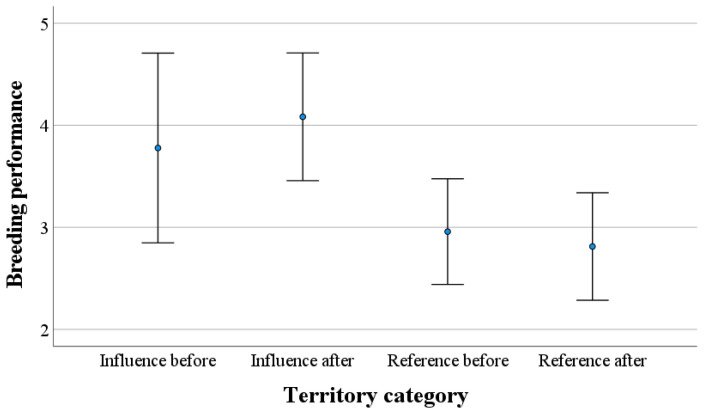
Breeding performance (score 1–6, see text) ± 2 SE for the 11 nests on Hitra and Frøya with at least one year with the production of young in the time period 2015–2021. The nests were within or outside an influenced area of 3 km from the closest wind turbine or power line before and after the disturbance started (*n* = 3 nests), or in reference areas further away (*n* = 8 nests).

The data to test hypothesis 2 were from only 11 nests on Hitra and Frøya, investigated yearly in the period 2015–2021, and with production of young for at least one of the years. A GLMM analysis with the breeding performance (values 1–6) as the target variable was statistically significantly higher inside than outside an influence distance of 3 km (coefficient = 2.244 ± 1.15, t = 1.953, *p* = 0.028). The other explanatory variables—year, island and disturbance or no disturbance for each year—were far from being statistically significant. However, this strange result that contrasted with our hypothesis was caused by a higher breeding performance within the influence area of 3 km already before the disturbance started, and the breeding performance did not change, neither in the influence areas nor in the reference areas when the disturbance started (Figure 3). The use of distance to the disturbance as an explanatory variable instead of influence area had a far-from-significant effect, as well as with other influence areas than 3 km.

## 4. Discussion

The present investigation focuses on the immediate response of territorial eagle owls to disturbances from the full construction period of wind farms and power lines, and a short period with active wind turbines and power lines. We found that territorial birds within 5 km from the disturbance left their territories to a significantly higher extent (41%) compared with the eagle owls in territories further away (23%). In addition, the distance from the nest site or the central part of the territory to the disturbance was significantly shorter for those territories that were abandoned compared with territories where the birds stayed. Our findings of the detrimental effects are in accordance with our prediction 1, and with other investigations on how eagle owls react on human disturbance [38,42,60].

The decline in eagle owl populations near the construction areas of wind farms and power lines might have three main explanations. Firstly, the increased mortality of eagle owls may have been caused by power lines and wind turbines. It is known by telemetry investigations that eagle owls fly more than 20 m above the ground 25% of the time [45], and that they can fly upwind like raptors high up in the air [18,61]. It is therefore reasonable that they are judged to be vulnerable to becoming killed by wind turbines [62], and eagle owls have been killed by wind turbines in several European countries [16]. It is also well-known that many eagle owls are killed by power lines [17,18,19,20,21,22]. Secondly, the desertion and avoidance of wind power areas by eagle owls caused by the noise and disturbance from the constructions. Several publications show the negative effects of noise and disturbances on bird populations (see Introduction). Thirdly, the possible indirect effects are that prey species of the eagle owl might die or leave the area, and that the eagle owl also leaves because less prey is available. Unfortunately, we do not know whether the abundance of prey was affected by the construction. Others have shown a positive correlation between the amount of prey and eagle owl population density [18,63]. The availability of prey is an important factor determining the density of breeding eagle owls, and it is unlikely that the shortage of nest sites limits its breeding density because of their flexibility in choice of nest sites [18]. Nest sites seem to be frequently available in the rocky environments that the eagle owls use as nesting sites in our investigation areas. Eagle owls might skip breeding in years with low food availability [64,65], and there is a positive relationship between territory occupancy and habitat quality [66]. Food availability is among the most important factors influencing fluctuations in eagle owl populations [18], and the occupation rate of eagle owl territories is found to be positively correlated with food availability and negatively correlated with mortality risk [67].

If one pair of eagle owl leaves the territory, there will be more space and less competition for the others. Inter-individual effects contribute to shaping space use and movement patterns in eagle owls [68]. Eagle owls seem to have considerable individual consistency in their movements with the repeated use of similar routes within their fixed home range, but might significantly change this route pattern between years, even if the same territory is occupied [69]. If a neighboring pair leaves their territory, remaining pairs can exploit a larger area without the restrictions caused by neighbors, and thereby breed equally or even more successfully. It is therefore possible that the negative effects of wind farm constructions might be easier to detect in the number of occupied territories than in breeding performance. This might be the reason why we did not find any negative effects on breeding performance after the constructions started compared with before, the opposite to our prediction.

We found that the breeding performance seemed to be better within the influence area of 3 km compared with the reference areas further away from the disturbances. However, this difference was present before the constructions started, and it did not change after the constructions started (Figure 3). The difference might be because eagle owls mostly hunt in open areas [37,70] in the same type of landscape used to construct wind farms. There were also few nests within the influence area of 3 km (*n* = 3) that could cause bias in the data. There were no statistically significant differences in breeding performance when we used distances of 4 or 5 km as the influence area, and we found no support for hypothesis 2.

There might be a time lag in population declines after disturbances if the eagle owls leave their territories after the most important prey species become less numerous. Eagle owls have a strong nest site fidelity [37], and the same breeding cliff in the middle part of Norway has been used for nearly 4000 years [47].

It is important to note that the disturbances in the present paper were in established eagle owl territories with little other human activity before the construction started. If there are appropriate nesting sites and a good food supply, the eagle owl can adapt to living closer to humans [43], and eagle owls can even breed in large cities [18]. This is also known in other eagle owl species, such as the Mackinder’s eagle owl (*Bubo capensis mackinderi*) in Africa [71], and the rock eagle owl (*Bubo bengalensis*) in India, which breeds in higher densities in highly human-altered landscapes that are richer in larger prey, such as rodents and birds [72].

In the present study, the nestling production we used was the number of young in the first half of June, when they were old enough to be ringed. Eagle owls in Norway suffered a high mortality rate that gradually declined during their first three years [73]. There might be several reasons for this high early mortality [18], and similar mortality patterns were found in other owl species [74] as well as in other bird species [75,76,77]. It is therefore uncertain how the results would be if we could follow the young for a longer period.

A lowered density in eagle owls might reduce the need for vocalizing among the remaining individuals, thus reducing the probability of being detected by passive auditory surveys such as sound meters [78]. Our experience in more than ten remote territories in our investigated area is that the territorial birds were detected by the use of sound recorders in all years. The eagle owls are not singing only to defend their territories, but also have intra-pair contact sounds uttered by both males and females [18].

There are many other possible threats to eagle owls [18], including pesticides, pollution [79,80], and mobbing corvids [37,38]. We believe that there should be no differences in the probability that eagle owls will leave their territories because of these factors; therefore, we assume that many changes in territory occupancy inside and outside the influence area can be described as wind farm and power line constructions. 

To our knowledge, no other study exists that shows the importance of the distance to various kinds of disturbances from eagle owl nests. However, there is some advice given by researchers to protect the eagle owl from disturbances, e.g., all building of houses and other disturbances should be at least 1 km from the nest site [81,82]. We found the highest impact of the disturbances on eagle owls when we used 4–5 km as the influence area. Fewer territories were within a 1–3 km distance from the closest wind turbine or power line, which gave a low statistical precision. However, more research is still needed to quantify the magnitude of human-related eagle owl mortality and its effects on the populations [18]. For the more effective conservation of eagle owls during different types of constructions, it might be interesting to know the disturbance contributions from each factor. In a wind farm and power line construction, that can be achieved by increasing human presence before the construction period starts to the same level that is expected during construction, then continue with the construction period, and thereafter activate the wind turbines. Before and after each step, the effects on eagle owls and prey abundance should be investigated during their breeding seasons. To be successful, there should be no delay in the effects of the different disturbance factors. The construction disturbance in our investigated area is finished, but the disturbance from rotating wind turbines and their sounds might also deter the eagle owls or their prey, which can be investigated in the ongoing study of these territories.

This is the first published survey in Norway that has investigated the short-term effects of establishing wind farms in or nearby the territories of eagle owls. The construction activities in the present study were performed throughout the year, including during the eagle owl breeding period, and were similar for the short period with active wind turbines. The eagle owls are stationary in the breeding area and were therefore continuously exposed to the disturbances for quite a long period. Despite not being very shy, at all times, the early breeding stages represent the most sensitive and fragile period for the eagle owl. If the female is disturbed at the nest during incubation or shortly after hatching, she might abandon the nest [18]. A conservation recommendation learned from the present investigation is that it should be investigated if an eagle owl is present in a construction area before the constructions start, and any eagle owl territories should affect how and when the construction can be carried out.

## 5. Conclusions

The effects of disturbance from the whole wind farm and power line construction period and a short period with active wind turbines were measured in 39 territories with eagle owls present before and/or after disturbances. More territories were abandoned within an influence area of 4–5 km compared with reference areas that were further away. Testing to discover if the influence area was shorter yielded no significant results, probably because there were few territories within the influence areas with shorter distances from the disturbances. The mean distance from the disturbances was shorter in the abandoned territories compared with the territories where eagle owls were observed both before and after the disturbance period. These results show that the eagle owl is vulnerable to anthropogenic disturbance in areas with little prior disturbance. This investigation is a contribution to a field with a lack of knowledge, and more investigations are needed to ensure a better conservation of this threatened bird species, and especially to find the real influence distance for different kind of disturbances.

## Data Availability

The data set is available from MH upon request.
